# Prion‐like proteins: from computational approaches to proteome‐wide analysis

**DOI:** 10.1002/2211-5463.13213

**Published:** 2021-06-17

**Authors:** Marcos Gil‐Garcia, Valentín Iglesias, Irantzu Pallarès, Salvador Ventura

**Affiliations:** ^1^ Departament de Bioquímica i Biologia Molecular Institut de Biotecnologia i de Biomedicina Universitat Autònoma de Barcelona Spain

**Keywords:** prion, prion‐like protein, functional amyloids, prion‐like prediction, bioinformatics, proteome screenings

## Abstract

Prions are self‐perpetuating proteins able to switch between a soluble state and an aggregated‐and‐transmissible conformation. These proteinaceous entities have been widely studied in yeast, where they are involved in hereditable phenotypic adaptations. The notion that such proteins could play functional roles and be positively selected by evolution has triggered the development of computational tools to identify prion‐like proteins in different kingdoms of life. These algorithms have succeeded in screening multiple proteomes, allowing the identification of prion‐like proteins in a diversity of unrelated organisms, evidencing that the prion phenomenon is well conserved among species. Interestingly enough, prion‐like proteins are not only connected with the formation of functional membraneless protein–nucleic acid coacervates, but are also linked to human diseases. This review addresses state‐of‐the‐art computational approaches to identify prion‐like proteins, describes proteome‐wide analysis efforts, discusses these unique proteins' functional role, and illustrates recently validated examples in different domains of life.

## Graphical abstract

Prions are proteins able to populate two conformational states, one of which is transmissible. The discovery of functional prions in yeast triggered the development of distinct computational approaches to discover prion‐like proteins in other organisms. This review delves into the rationale behind these algorithms and discusses the new insights gathered from proteome‐wide data analysis in different kingdoms of life.

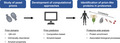

**Abbreviations**
IDR: intrinsically disordered region
LCR: low‐complexity region
LLPS: liquid‐liquid phase separation
PrD: prion domain
PrLD: prion‐like domain
RBD: RNA‐binding domain
RNP granules: ribonucleoprotein granules

In recent decades, our perception of the prion phenomena has evolved remarkably. Initially considered strictly as infectious proteinaceous elements linked to mammalian neurological disorders, prions and prion‐like proteins are now known to play critical roles in regulating evolutionary conserved biological processes [1, 2].

Prions are a particular subset of proteins able to adopt different conformational and functional states, where at least one of them is amyloidogenic and transmissible, templating homologous polypeptide sequences, even across species [3, 4]. These biomolecules show the ability to aggregate into multiple different amyloid structures, that is, strains, with distinct physicochemical properties and propagative features [5, 6]. Most of our knowledge on these polypeptides' self‐replicating properties comes from the study of *Saccharomyces cerevisiae* prion proteins, where the transition from a soluble conformation to an aggregated state has been related to heritable phenotypic changes [7, 8, 9, 10]. In most yeast prions, but not all, the prion activity is encoded in a fraction of the protein, the prion domains (PrD) that share a set of compositional features since they correspond to low‐complexity regions (LCRs), with enrichment in glutamine (Q) and asparagine (N) and depletion in charged and hydrophobic residues. They also share conformational traits as they are embedded in long intrinsically disordered regions (IDRs). These PrDs are both necessary and sufficient for prion conformational conversion [11, 12, 13, 14].

Classical amyloid prediction algorithms are blind to the amyloidogenic potential of Q/N‐rich stretches, suggesting that the sequence‐specific physicochemical properties that drive the conformational promiscuity of prions do not conform to the standard features that govern the formation of canonical amyloid aggregates (e.g., high hydrophobicity and intrinsic β‐sheet propensity). The extensive research devoted to the characterization of the PrDs of the yeast prions Sup35 and Ure2p (their prionogenic states are known as [PSI+] and [URE3], respectively) has enabled to dissect the different molecular determinants behind prion conversion [15, 16, 17, 18]. Collectively, this knowledge has fueled the development of a collection of bioinformatics tools to identify proteins bearing similar prion‐like domains (PrLDs) in biological relevant sequence databases as well as in complete proteomes [13, 14, 19, 20, 21, 22, 23, 24, 25, 26, 27].

The implementation of computational proteome‐wide analyses has identified thousands of new proteins potentially harboring PrLDs in a wide variety of organisms, ranging from viruses to humans [13, 21, 28, 29, 30, 31, 32, 33, 34, 35]. Thus, supporting the idea that prions, and their evolutionary conserved capacity to experiment conformational transitions, are exploited by cells as important functional elements. Although the prionic load varies significantly among organisms, accounting for < 1% of human proteins or up to 10% in *Plasmodium falciparum* [31, 33, 36], they seem to conserve functional and structural traits. Collectively, prion‐like subproteomes consist of modular proteins containing multiple functionally associated domains, with a particular abundance of RNA‐ and DNA‐binding motifs [37, 38]. In this context, it is becoming increasingly evident that PrLDs are involved in highly connected interaction networks and associated with the formation of dynamic membraneless intracellular compartments through liquid‐liquid phase separation (LLPS) [39, 40, 41]. Indeed, the presence of structurally disordered regions in proteins correlates with the propensity to establish weak–transient interactions that drive liquid‐to‐solid phase transitions [38, 42]. Remarkably, in the human proteome, the PrLD subset includes well‐characterized prion‐like proteins such as FUS, TDP‐43, TIA1, or hnRNPA1. Mutations in these proteins are associated with neurodegenerative disorders, and they often map at their PrLDs, promoting the conversion of the condensate state into solid amyloid‐like aggregates [43, 44, 45].

Despite significant progress in our knowledge of the prion phenomenon, both in beneficial cellular processes and pathological conditions, our understanding of the challenging PrLDs biology would benefit from the global view provided by large scale analysis. We describe a collection of computational approaches that exhibit significant distinctive features, despite being all trained on yeast prions sequences. The application of these algorithms to proteome‐wide analyses has led to identifying relevant PrLDs in organisms of divergent biological complexity. We discuss their involvement in cellular regulatory functions, their role as molecular tools for adaptation, and their linkage to disease. Overall, predictors have allowed the discovery of PrLDs functions that would have never been unveiled analyzing individual proteins.

## Computational tools for the prediction of prion‐like domains

In recent years, and especially since next‐generation sequencing techniques became available, annotated sequences on public databases have exponentially increased. In this context, large‐scale bioinformatics analysis should allow screening for prion‐like proteins across organisms of distant clades. However, conventional aggregation prediction algorithms often fail to discriminate prions from nonprions correctly [27, 46, 47] (Table 1).

**Table 1:** Performance of aggregation prediction methods when identifying prionic sequences. AGGRESCAN [132], PATH [133], RFAmyloid [134] and AmyloGram [135] prediction methods were run with default parameters and results were obtained using their standard thresholds. PrionW [19] is intended for predicting prion‐like proteins and was used for performance comparison (in italics). The sensitivity, specificity, precision, accuracy, Matthews correlation coefficient (MCC) and F1 Score were calculated from yeast prion domains and prion‐like domains experimentally validated by Alberti and coworkers [13]. The authors characterized the domains for their amyloid and prion forming ability in four assays and scored them from 0 to 10. As described previously [26], those domains that were positive in all four assays and scored ≥ 9 were considered prions, and nonprions those sequences scoring ≤ 2 and being positive in one assay at maximum. The dataset was composed of 12 true positives (TP) (including *bona fide* prions Sup35, New1, Swi1, Ure2p, Rnq1) and 39 true negatives (TN). False negatives and false positives are abbreviated as FN and FP, respectively.

Algorithm	TP	TN	FP	FN	Sensitivity	Specificity	Precision	Accuracy	MCC	F1 score
*PrionW*	*11*	*37*	*2*	*1*	*0.92*	*0.95*	*0.85*	*0.94*	*0.84*	*0.88*
AGGRESCAN	0	39	0	12	0.00	1.00	–	0.76	–	0.00
PATH	3	39	0	9	0.25	1.00	1.00	0.82	0.45	0.40
RFAmyloid	8	15	24	4	0.67	0.38	0.25	0.45	0.04	0.36
AmyloGram	10	11	28	2	0.83	0.28	0.26	0.41	0.11	0.40

Therefore, there was a need for new programs that exploit the specific features of PrD in their predictions. Two different models were proposed for decoding the forces driving conformational changes from primary sequences. The compositional model considered that it would be necessary a large number of weak interactions distributed along the PrD for which a biased amino acidic composition was required [27]. The soft‐amyloid model relied on the presence of stretches of mild amyloid potential, where the amyloid load is more diffuse than in ‘classical’ pathogenic amyloids, embedded within an intrinsically disordered PrD [26, 48]. Several groups have approached prion‐like prediction applying these different, but as we will see, complementary perspectives, resulting in multiple algorithms (Table 2) [13, 14, 19, 20, 21, 22, 23, 24, 25, 26, 27]. This section will review six representative state‐of‐the‐art bioinformatics tools, which have had a significant impact on identifying novel prion‐like domains and their associated proteins.

**Table 2:** Prion‐like prediction methods and applied analytical rationale.

Algorithm	Strategy	Brief description/Underlying rationale	Availability	References
DIANA	Compositional	Identifies Q/N‐rich domains by counting Qs and Ns in 80 consecutive amino acids windows and retrieves the most enriched stretch above a 30‐Q/N threshold. Stretch length and minimum Q/Ns content are based on the length and Q/N percentage of Sup35 and Ure2p yeast prions and human disease‐causing polyQ‐expansions	–	[25]
LPS	Compositional	The program is designed to retrieve any compositionally biased sequence, and it has been used to identify Q/N‐rich regions as a proxy to PrLD. It first searches for all possible single amino acid bias by comparing each window against the inputted background frequencies and retrieving the lowest probability stretch. Subsequent updates allowed automatic calculation of bias for multiple residue types by checking if their combined probability was lower than those of the individual residues separately. LPS calculates for and against biases	Script	[23, 136]
PAPA	Compositional	Prediction is made on disordered segments, exploiting an amino acid propensity scale obtained by random mutating a short stretch of a prionic Sup35 variant	Web server + script	[14, 27]
PLAAC	Compositional	Applies an HMM trained on 28 yeast PrD and PrLD with high experimental prion propensity against user‐selected backgrounds of amino acid frequencies	Web server + script	[13, 24]
pWALTZ	Amyloid	Applies the WALTZs' experimentally‐derived amyloid propensity scoring matrix to longer stretches, averaging the amyloid load over 21‐residues stretches and retrieving the strongest amyloid‐core	Executable	[26, 137]
PrionW	Compositional + Amyloid	Disordered fragments with a minimum QN content are evaluated with pWALTZ. The QN‐threshold can be adjusted for different species' background frequencies	Web server	[19]
PrionScan	Compositional	Uses an unsupervised classifier and a statistical representation of PrLD relying on the amino acid frequencies of positive and negative sequences in Lindquist's dataset. PrionScan incorporates a built‐in database that regularly updates its predictions for UniProt KB releases	Web server + Database	[21, 22]
pRANK	Compositional/Machine learning	Implements a supervised learning strategy, trained on 22 known Q/N‐rich yeast PrD as positive sequences and the remaining proteome with < 90% similarity to them as negative ones	Web server + script	[20]

### Composition‐based predictors

Several lines of evidence, including the presence of Q/N‐rich domains in yeast prions that form amyloids (and their inactivation upon mutation of Q and N residues) [49], Q‐repeats in aggregating proteins involved in human disorders [25], or the experimental demonstration that inter‐chain hydrogen bonding between glutamines stabilizes antiparallel β‐strand conformations [50], indicated that these amide‐carrying amino acids played an important role in the self‐propagation of aggregated species [25, 49]. These observations lie behind the compositional model for prion‐like conversion, according to which many weak, sequence‐independent interactions across a sizeable low complexity region would drive the structural conversion into an amyloid aggregate [27]. Different algorithms have been developed following this principle. They essentially differ in the contribution assigned to each of the 20 proteinogenic amino acids.

The identification of a scrambled version of Sup35 yeast prion that undergoes prion conversion without overexpression allowed Toombs *et al*. to design a strategy to quantify each amino acid's influence on this process. They mutated an 8‐amino acid segment of this Sup35 version randomly and sequenced those clones that kept the parental prionic behavior. The prion propensity was then defined as the log‐odds ratio of each amino acid frequency among the prion‐forming clones, relative to the starting library [14]. The method worked better on top of predefined disordered regions (for which they applied the FoldIndex algorithm [51]) and smoothing the scores using 41‐residue windows. The resulting algorithm was made available through a web server and a python code as Prion Aggregation Prediction Algorithm (PAPA) [27] and used to design synthetic prions that maintained the Sup35 Q+N content. In a follow‐up study, Cascarina and Ross updated PAPA by modifying the minimal PrLD length and lowering the requirements for positive detection in order to study the influence of genetic variation, mutations, or the effect of post‐transcriptional and post‐translational modifications on putative prion‐like proteins; in an attempt to understand how protein variation could modulate the prion‐like outcome [52], an effect previously demonstrated for the human hnRNPDL prion‐like protein [53].

Lindquist and coworkers applied another strategy to unravel the determinants behind prion conversion. They trained a hidden Markov Model (HMM) on PrD detection using the four *bona fide* yeast prions known at that time and ranked with it the entire yeast genome. The highest 100 predicted prion domains were tested for aggregation, amyloidogenicity, and the ability to maintain the prion phenotype on a chimeric Sup35, which allowed them to discover a new real prion, Mot3. The 28 domains, which scored highest in those experiments, were later used to feed the HMM. This tool was made available as the prion‐like amino acid composition (PLAAC) prediction algorithm in the form of a web server and a java code. PLAAC incorporates the possibility of adjusting the HMM calculations with either a set of prepopulated species background or the entire input sequences. The algorithm also incorporates PAPA, DIANA, and FoldIndex calculations and returns their outputs.

Afsar Minhas, Ben‐Hur and Ross further approached the phenomena from a machine‐learning perspective. Two main barriers hamper the use of neural networks to develop a binary prion classifier: (a) the low number of annotated *bona fide* PrDs and (b) the annotated PrDs usually entail an area larger than the effective sequence required for prion conversion. They addressed these limitations using a supervised learning method known as multiple‐instance learning (MIL), which allows modeling with some degree of uncertainty, while using sequence composition alone as an input. The method, named prion RANKing and classification (pRANK), was trained on top of 22 known Q/N rich yeast PrDs in the Lindquist's dataset and released as a web server and a python code [20]. pRANK showed an excellent discrimination performance on top of the yeast proteome. However, the high weight assigned by pRANK to Q results in an inherent bias to detect false positives arising from polyQ stretches, something that should be regarded with care when working with strongly compositionally biased proteomes.

Ventura and Sancho evaluated Lindquist's top 29 PrLD dataset and established their amino acidic frequencies. Contrasting their composition with other high scoring but nonprion Q/N‐rich PrLD sequences from the same study, they built up the predictive approach PrionScan. This algorithm revealed a positive bias toward Q, N, S, and Y residues and a negative one toward charged residues, as well as C and W, in well‐performing prion‐like sequences. PrionScan was applied to screen the whole UniProt KB rendering notable differences for organisms of different taxonomic categories; and identifying individual proteomes with a high prionic load, such as the ones of *Dictyostelium discoideum* and *P. falciparum* with 20% and 10% of predicted sequences, respectively. PrionScan was made available through a web server, a Perl code, and a built‐in database. For updated predictions, PrionScan regularly analyses UniProtKB database releases and stores them into its built‐in database, currently holding > 28 000 prion candidates. Over the years, several groups have applied prediction methods to generate online databases for their prion‐like candidates [54, 55], but by the time of this writing, only PrionScan was currently active.

### Soft amyloid‐based predictors

Scrambled Sup35 and PrDs can adopt prionic states; however, their propensities differ from that of the respective wild‐type forms, indicating that sequential traits modulate conversion, which is consistent with the in‐register parallel β‐sheet structure proposed for their amyloid states [56, 57], still conventional amyloid predictors were blind to the sequential determinants of such amyloidogenicity. Ventura and co‐workers realized that these yeast PrDs had a lower amyloid propensity and that it was diluted into more amino acids than in classical amyloids [26]. These made them postulate the soft‐amyloid model, in which a short linear stretch of softer amyloid propensity inside an IDR would kick start the conformational switch toward the aggregated, amyloid state [26]. They used the position‐specific amyloid predictor WALTZ and extended its length to 21 residues based on this being the minimum length of a transmissible β‐fold at that time (as seen in the atomic structure of HET‐s prion from the fungus *Podospora anserina*). This approach, named pWALTZ, was tested on top of Lindquists' PrLD dataset, showing higher discriminatory potential than PAPA. Moreover, it identified amyloid cores in PAPA synthetic prions and disease‐related mutations in human prion‐like proteins and the lack of them in PAPAs' nonprion engineered sequences [26]. pWALTZ was made available as an executable file.

As pWALTZ was trained on top of yeast PrD, it was not designed for detecting IDR or PrLD on full protein sequences but relied on users inputting putative prion‐like domains. As such, its predictive power was expected to decrease when applied in proteomic scale screenings, as it could mistakenly detect as soft‐amyloids regions belonging to the hydrophobic core of folded proteins or membrane‐associated proteins. The same group applied pWALTZ on defined sequence contexts in a novel PrLD predictor to overcome these limitations. This software, which was named PrionW, defines the PrLD by first applying disorder calculations (by deploying FoldIndex predictions) and secondly calculating Q/N‐richness on top of those IDRs [19]. It allowed users to specify the minimum Q/N requirement for their sequences to be considered prion‐like, which addressed species‐specific background compositional bias. PrionW selects the IDR with the highest number of Q/N and passes it to pWALTZ, where the highest‐scoring soft‐amyloid core is selected. PrionW was made available as a web server.

### Predicting prion‐like proteins in complete proteomes

Prediction methods like PLAAC, PAPA, and PrionScan have succeeded in identifying prion‐like proteins by comparing the amino acid compositional similarity of protein candidates to the average composition of *bona fide* yeast prions [13, 14, 21, 22, 27, 28, 29, 58, 59]. On the other hand, pWALTZ and PrionW algorithms, which consider in their predictions prion amyloid propensity, have also shown to be highly accurate for discriminating yeast prions [19, 26], and they have been applied not only for the characterization of large datasets [32, 33, 60] but also for the discovery of soft‐amyloid cores in yeast prions [61]. Further studies identified the one in Sup35 as the region required for the induction, propagation, and inheritance of the prion state in mammalian cells [62].

**Fig. 1:**
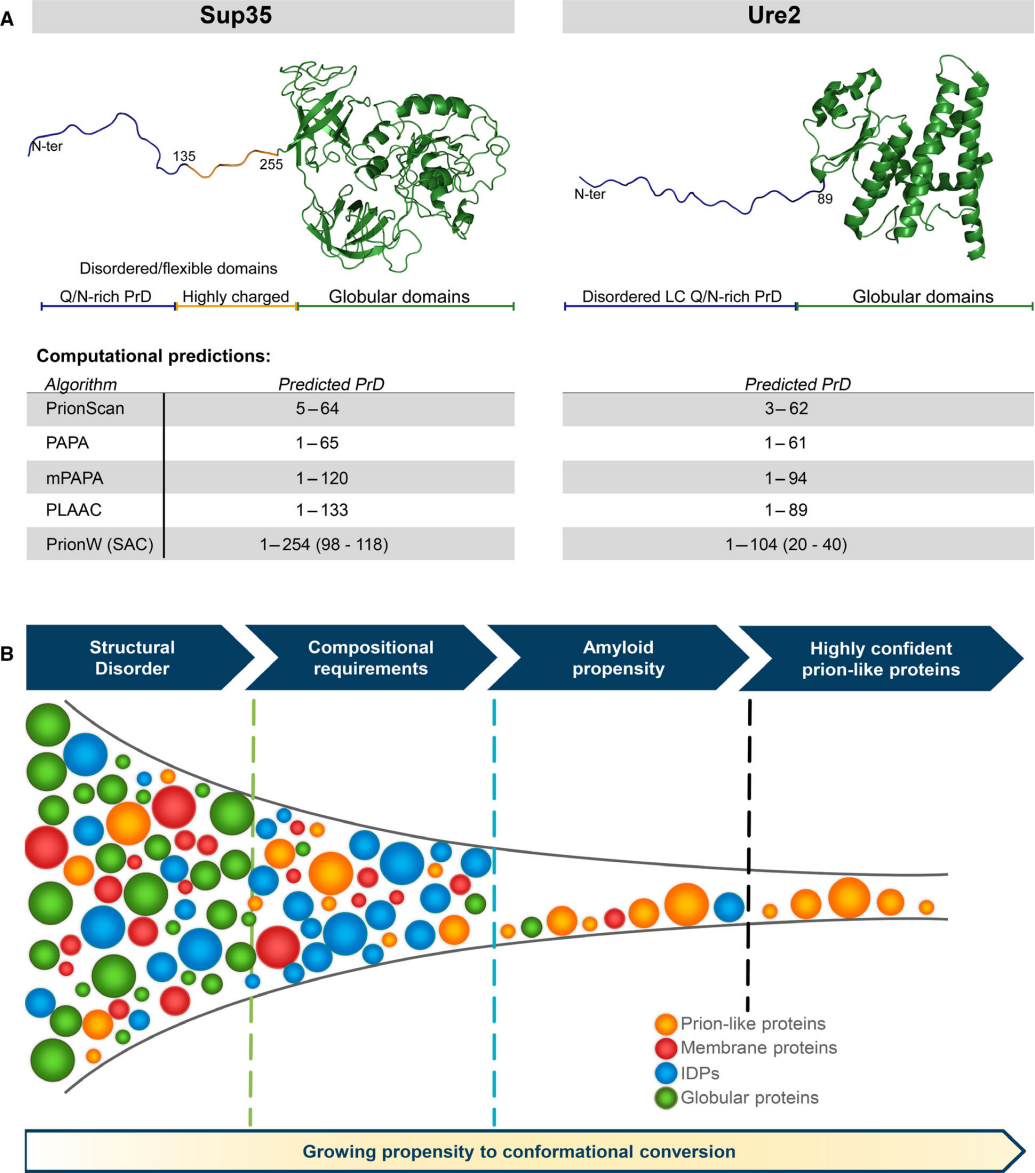
Bioinformatics has been a valuable tool in the discovery of prion‐like proteins. (A) Prion‐like prediction methods correctly identify PrD in yeast prion proteins Sup35 and Ure2p. Disordered N‐terminal regions shown in cartoon representation are models not derived from structural data. (B) Proposed pipeline for optimizing prion‐like proteins discovery. Large scale computational analysis offers a powerful alternative to time‐consuming and more expensive experimental approaches. Stepwise sequential restrictions in the selection from a pool of initial candidates increase the success rate for discovering novel prion‐like proteins. (a) mPAPA corresponds to the 2020 modified version of PAPA (b) pWALTZ requires a previously defined PrLD; thus pWALTZ soft‐amyloid core predictions are only shown for PrionW.

Regardless of the relative contribution of each of the above‐discussed features to prion‐like conversion, a combination of compositional and soft‐amyloid requirements increases the stringency of the prediction, thus increasing the confidence in predicted candidates' performance (Fig. 1). Remarkably, this combinative strategy led to identifying the transcription terminator Rho factor as the first prion protein in bacteria [30, 60, 63].

Of note, the interplay between compositional bias and the presence of short‐amyloid stretches has enabled predicting mutations impact on the aggregation propensity of prion‐like proteins [64, 65] in various diseases related to mammalian prion‐like protein variants [66, 67, 68, 69].

## Proteome‐wide analysis of prion‐like proteins in different kingdoms of life

The study of prions in baker's yeast has been pivotal to understand prion properties and their associated‐functions [70, 71]. The yeast HMM‐predicted subproteome presented an enrichment in proteins involved in gene expression processes (transcription factors and RNA‐binding proteins) consistent with their role as epigenetic devices of adaptation to stress [13].

By definition, canonical prions exhibit QN‐rich domains but also the ability to populate amyloid supramolecular conformations and a Hsp104‐dependent cell‐to‐cell propagation [72, 73]. Although it is not the scope of this review, it is worth to mention that certain proteins lacking these canonical traits may act as prions, including the so‐called mnemons, which can suffer conformational switches, but cannot propagate to daughter cells, acting as a kind of cellular memory [74, 75].

**Fig. 2:**
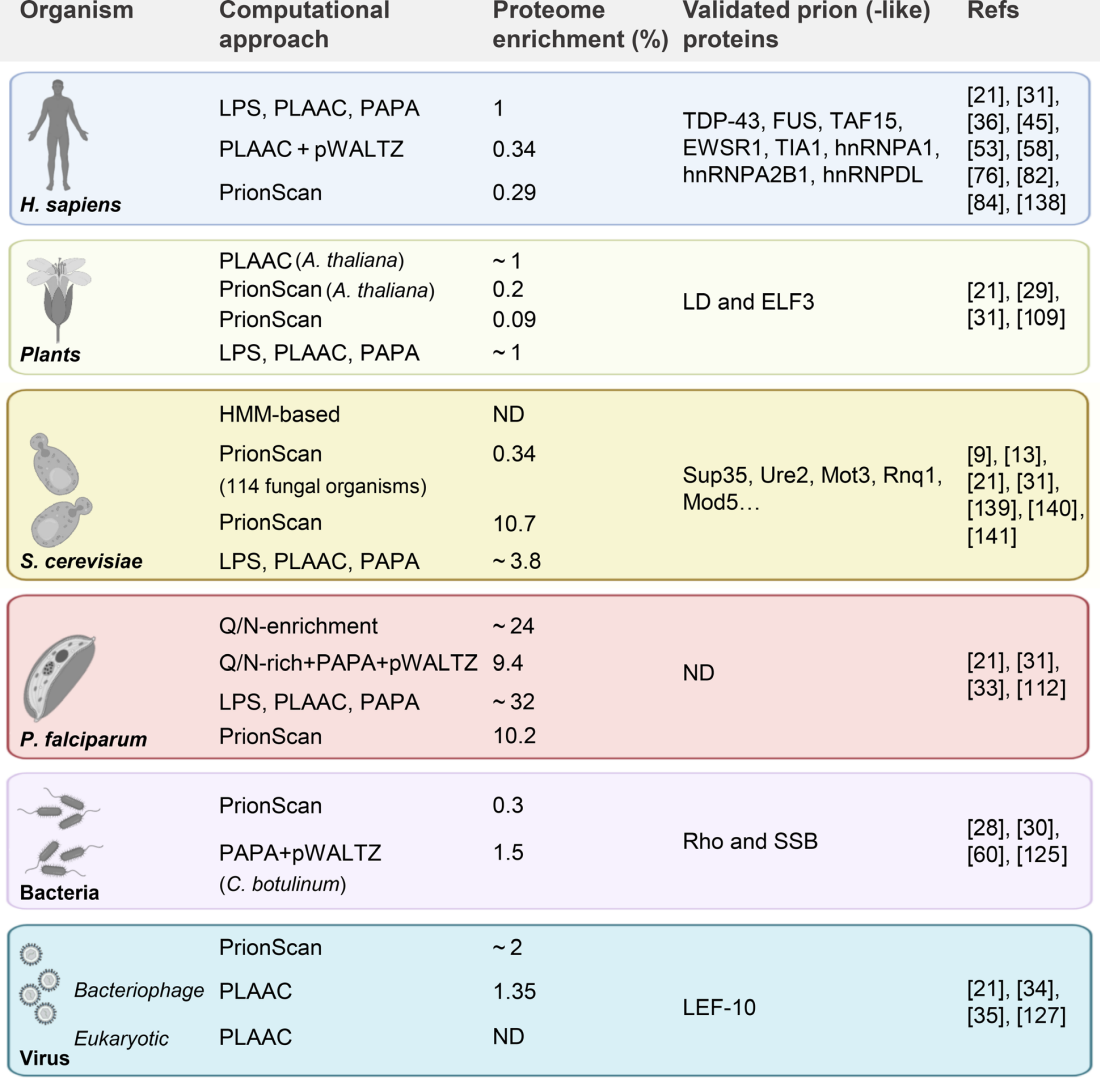
Proteome‐wide analyses of prion‐like proteins in distinct kingdoms of life. Large‐scale analyses approximate the content of PrLD‐containing proteins in the proteomes unrelated organisms. Experimentally‐validated examples (if any) of prion‐like proteins and proteome enrichments are indicated for each species. ND, not determined. Created with BioRender.com.

The success in the computational identification of previously undescribed canonical yeast prions has encouraged the discovery of similar proteins (generically termed as prion‐like proteins) in a wide variety of organisms, ranging from virus to higher eukaryotes. In next sections, we review the most recent proteome‐wide analysis aimed to identify such prion‐like proteins, delving into their biological functions, and providing data on experimentally validated examples (Fig. 2).

### The human proteome

The connection between FUS and TDP‐43, two proteins bearing a PrLD together with RNA recognition motifs (RRM), and neurological diseases suggested a connection between this kind of architecture and pathology [37]. RRM is one of the most versatile RNA‐binding domains (RBDs) and is conserved across different kingdoms of life. Gitler and coworkers used a yeast functional screen, together with bioinformatics analysis, on top of the RRM‐containing human subproteome to identify new putative prion‐like proteins behind neurological disorders [76]. They obtained evidence of a PrLD‐enrichment in RNA‐binding proteins and revealed the association of yet another RNA‐binding protein, TAF15, with neurological damage.

One successful attempt to computationally identify PrLD‐containing proteins in humans was performed by An and Harrison [31]. In this study, the authors selected candidates using three different prediction methods (LPS, PLAAC, and PAPA), uncovering that they account for approximately 1% of the human proteome. A subsequent analysis of the data corroborated a statistically significant relation between human prion‐like proteins and neurological diseases. However, the analysis also indicated that these proteins' expression was not exceptionally high in the central nervous system.

Ventura and coworkers applied a more stringent procedure to identifying human prion‐like proteins [36]. The prediction scheme consisted of an initial screening for the presence of regions displaying compositional similarity to yeast PrDs using PLAAC, followed by a refined search of soft amyloid stretches using pWALTZ. The collected results revealed a total of 242 proteins harboring PrLDs from an initial dataset of > 70 000 proteins, accounting, in this case, for 0.34% of the human proteome.

An in‐depth analysis of these PrLD‐containing proteins' biological role evidenced a significant enrichment in RNA and DNA associated processes, particularly the ones related to positive regulation of transcription from RNA polymerase promoter, mRNA splicing, and RNA processing. Analogously, the most enriched functions were nucleic acid binding and transcription, and the cellular components overrepresented in this subproteome were nuclear structures, including the nucleus itself and the ribonucleoprotein complex. Not surprisingly, the most abundant Pfam cluster associated with PrLDs corresponded to DNA‐ and RNA‐binding domains such as the RRM domain. Therefore, even if the Gitler and Ventura laboratories departed from different premises and approaches, their studies converged to show an association between PrLD and RRM moieties. Overall, the predictions were consistent with evidence from the literature that reported a crucial role of human prion‐like proteins in forming ribonucleoprotein (RNP) granules involved in the post‐transcriptional regulation of gene expression.

An analysis of the expression profiles of the 242 protein's in Ventura's dataset revealed that, in agreement with An and Harrison observations [31], the expression of these polypeptides was not restricted to the nervous system but instead distributed among different tissues, suggesting that, contrary to what was initially thought, they could be involved in diverse functions across the human body. For example, the Mediator complex, a transcriptional coactivator required for gene transcription by RNA polymerase II in eukaryotes [77], contains subunits displaying PrLDs in their sequences. Many of the identified proteins seemed to exert essential functions, which immediately suggested that alterations in their activities could trigger the development of multiple diseases [78].

Indeed, a very robust association between human prion‐like proteins and disease was evident in this study. Notably, as expected from their broad tissue distribution, these polypeptides appeared to be involved in neurological problems, but also in different types of cancer and viral infections. The reasons behind this tight relationship likely rely on (a) the high connectivity of prion‐like protein‐protein interactions networks and (b) the inherent aggregation propensity of PrLDs, in such a way that the establishment of promiscuous non‐native and irreversible intermolecular interactions would unfailingly disrupt network fluxes and impact cell viability.

The best‐characterized examples of disease‐associated prion‐like proteins are the TDP‐43 and FUS proteins, as mentioned above [79, 80]. TDP‐43 was identified as the main component of protein aggregates in patients with amyotrophic lateral sclerosis (ALS) and some types of frontotemporal lobar degeneration (FTLD) [81]. TDP‐43 possesses two RRMs and a C‐terminal PrLD, where most of the disease‐associated mutations map [82]. In FUS, the PrLD is located at the N‐terminal extreme, and there is only an RRM [83, 84]. Its aggregation is linked to ALS and FTLD, and mutations in its sequence are involved in disease, especially those occurring at its PrLD and in a C‐terminal segment predicted as a nuclear localization signal [37, 82]. Other RNA‐binding proteins such as EWSR1 and TAF15 are also involved in ALS and FTLD; however, their disease‐associated mutations are placed outside PrLDs and respond to functional domains inactivation [58, 85].

Besides their invariable connection with pathological phenotypes, proteins harboring PrLDs have not been evolutionary purged out. In that context, a growing body of evidence has demonstrated that such domains are indispensable for forming membraneless compartments where RNA and proteins are recruited in response to diverse cellular and environmental signals [86, 87]. These dynamic structures are formed through a process commonly known as liquid‐liquid phase separation (LLPS) that involves establishing weak and noncovalent transient interactions between prion‐like proteins [41, 86, 88]. Although the ubiquity of PrLD‐containing proteins in membraneless compartments suggested that these domains are necessary and sufficient to promote LLPS [89, 90], recent results have demonstrated that interactions between residues located at PrLDs and RBDs are essential to trigger the formation of such protein condensates in vivo [91]. What is clear is that the deregulation of this process can promote an aberrant irreversible transition toward a more‐solid, aggregated, and in some cases, amyloid‐like state associated with the onset of devastating proteinopathies. It seems that both loss and toxic gain of function are at the core of these pathologies [10, 43, 45]. LLPS deregulation can be caused by changes in protein concentration, evoking the establishment of more frequent intermolecular interactions, or by point mutations that strengthen LLPS interactions and increase the kinetic barrier for dissociation, altering the material properties of the condensates. The result is, in both cases, less dynamic and thus dysfunctional condensates. In this context, there is a high interest in new therapeutic approaches that prevent or restore aberrant phase transitions [92, 93].

Cells exploit different stratagems to alter the assembly properties of PrLD‐containing proteins. One of these strategies is the process of alternative splicing (AS), a molecular event particularly relevant in human prion‐like proteins [94, 95]. Batlle *et al*. [53] have demonstrated that the self‐assembling properties of the hnRNPDL prion‐like protein are strongly influenced by its AS. The three different hnRNPDL‐isoforms generated by AS clearly differ in their propensity to LLPS, with one forming liquid droplets at very low concentrations, whereas another one is LLPS‐incompetent. The control of assembly by AS also occurs in the prion‐like proteins of other organisms, like in *Drosophila* Orb2/CPEB, where it regulates self‐templating at the synapse, which is correlated with the facilitation of long‐term memory [96].

Not only the final primary sequence of the polypeptide is relevant for its condensation potential, but this property is strongly impacted by the post‐translational modification of residues that contribute the most to the dynamics of condensates, including arginine methylation or tyrosine and serine phosphorylation [97, 98]. In this way, hypomethylated FUS has been found in insoluble aggregates of FTLD patients, suggesting that deficient arginine methylation contributes to this pathological phenotype [99, 100].

### The plant proteome

Plants cannot move from one place to another the way animals can. However, they quickly react to their surroundings and adapt their physiological response to ensure proper development and survival. Some of these adaptations seem to be regulated by amyloid and/or prion‐enciphered events [29, 101, 102].

The first detailed biocomputational analysis of prion‐like proteins in plants was conducted by Chakrabortee *et al*. in the *Arabidopsis thaliana* proteome. Applying the compositional algorithm PLAAC, they identified 474 putative proteins bearing PrLDs, accounting for roughly 1% of the proteome [29]. Ontology analysis revealed an enrichment in biological processes related to gene expression and regulation as well as in RNA‐binding. These enriched processes and the associated molecular functions are common to those reported for the human proteome and, as we will see below, to the ones identified in the rest of analyzed proteomes, independently of the taxon of origin, something that ultimately arises from the fact that prion‐like proteins are inherently modular and, as described, PrLDs tend to associate with RNA‐ or DNA‐binding domains.

In *A. thaliana*, the predicted PrLDs were also connected to plant‐specific biochemical pathways such as those involved in reproductive and flower development processes. Four proteins involved in autonomous flowering exhibited predicted PrLDs, but only the Luminidependens protein (LD) recapitulated some of the classical characteristics of prion proteins when it was expressed and characterized in yeast.

The LD‐PrLD aggregates and can replace the Sup35‐PrLD in yeast promoting the characteristic phenotypic switch; however, the self‐perpetuating conformation corresponds to low molecular weight oligomers, instead of high‐molecular‐weight amyloid fibrils. Also, contrary to most classical yeast prions, LD‐PrLD propagation is not influenced by Hsp104 and might depend instead on Hsp70 or Hsp90 activities, a phenomenon described for the noncanonical fungal prions [GAR+, ESI+ and SMAUG+] [103, 104, 105, 106]. LD protein bears a DNA‐binding homeodomain able to regulate transcriptional events [107]. It was proposed that LD self‐assembly could alter its activity and evoke chromatin modifications involved in flowering decisions, representing an epigenetic memory mechanism previously undescribed in the plant realm.

More recently, Jung *et al*. have identified EARLY FLOWERING 3 (ELF3), a component of the evening complex related to the plant circadian clock that undergoes prion conversion, functioning as a thermosensor in *A. thaliana* [108, 109]. ELF3 harbors a polyQ‐PrLD able to transit between a soluble and a phase‐separated state depending on the environmental temperature. *A. thaliana* exploits the self‐assembling properties of ELF3 as a thermosensory mechanism to promote the plant growth and flowering at specific temperatures. Jung and co‐workers revealed that ELF3‐PrLD length varies across plants species living in different climates, suggesting that this divergence could be the product of an evolutive mechanism for the optimization of thermal responsiveness according to the environmental conditions [110]. This strategy, and in general prion switches, exploit the biophysical properties of polypeptides to regulate their conformational states without requiring a dedicate machinery for post‐translational modifications, resulting in a rapid and intuitive physiological response to temperature fluctuations.

Although great strides have been made in the study of PrLDs in plants, much remains to be understood and we envision that significant prion‐based molecular processes wait to be discovered in this kingdom.

### The *Plasmodium falciparum* proteome

The protozoan parasite *P. falciparum* is the primary cause of severe malaria and responsible for approximately half‐a‐million annual human deaths (World Malaria Report, November 2020) [111]. Comparative genome analyses revealed an exceptionally high AT content compared to other eukaryotes, with nearly 80% in coding regions. This is translated into an unusual proteome where the 30% corresponds to LCRs highly enriched in asparagine residues [112]. Low complexity and N‐enrichment are two of the characteristics of PrLDs, which already suggested that *P. falciparum* might be especially rich in prion‐like proteins.

Singh *et al*. [112] performed the first computational survey for PrLDs in *P. falciparum*, which they defined as consecutive stretches of 80 residues that contain at least 30 Q and/or N, unveiling that the 24% of *P. falciparum* proteins harbor at least one of those Q/N‐rich domains. Considering the inherent aggregation propensity of PrLDs, it is intriguing how organisms like *P. falciparum* or *D. discoideum* can stand with very high prionic loads. Detailed analyses of their proteomes have revealed an adapted protein quality machinery (e.g., PfHsp110c and SRCP1 proteins in *Plasmodium* and *Dictyostelium*, respectively) to keep their highly aggregation‐prone proteome under control and ensure the fitness these species in their natural environments [113, 114, 115].

To further characterize *P. falciparum* prion biology, Pallarès *et al*. [33] applied a more stringent biocomputational approach. First, as in Singh *et al*., LCR enriched in Q/N residues were identified. Subsequently, these regions were evaluated with the PAPA algorithm, and the positive sequences were scanned with pWALTZ. Thus, the predicted PrLDs were Q/N rich, intrinsically disordered, similar in composition to yeast PrDs, and with a cryptic propensity to assemble into amyloid‐like conformations. This restrictive strategy rendered a total of 503 PrLD‐containing proteins. This represents 1/3 of those found in composition only based predictions; however, they still represent 9.4% of the proteome [33], confirming that, effectively, one of the consequences of the *P. falciparum* genome bias is a prion‐like enriched proteome.

Functional analysis of this curated subproteome provided interesting insights into prion‐like proteins' role in this deadly parasite [33]. Again, the most enriched biological processes included gene expression and transcription regulation, reinforcing a preserved functional role of PrLDs across all kingdoms of life. More interestingly, the analysis also revealed significant associations with specific *P. falciparum* processes not identified in previous proteome analyses, including protein localization and regulation of vesicle‐mediated transport. This is important because the traffic of parasite proteins across the host cell plays a key role in host‐parasite interactions, pathogenesis, and disease susceptibility [116]. Between the prion‐like constituent functional domains, the most enriched ones were those related to nucleotide‐binding, including the ubiquitous RRM, but also domains not detected in other organisms such as the ApiAP2 domain. Indeed, 50% of the ApiAP2 protein family was shown to bear a PrLD. These proteins are the central transcriptional regulators in Plasmodium parasites and the other Apicomplexa [117], playing crucial roles in gametocyte maturation [118], organ infection [119], and the development of host immunity [120]. Thus, the analysis uncovered the association of putative PrLDs with essential functions connected with Plasmodium life cycle regulation and its adaptation to the host. Additionally, the authors demonstrated experimentally that these PrLDs contained sequences able to spontaneously self‐assemble into amyloid‐like structures, which can potentially trigger protein conformational conversion. Although no *bona fide* prion protein has been experimentally validated in *P. falciparum* yet, their predicted abundance and the fact that, contrary to what was initially thought, they do not correspond to spurious sequences but instead seem to exert important and well‐conserved functional roles in the parasite, might offer a new class of therapeutic targets to treat malaria.

### The bacterial proteomes

Bacteria are single celled microorganisms adapted to multiple and diverse environments. The evidence that eukaryotic unicellular organisms exploit prion mechanisms to adapt in front of environmental fluctuations and the discovery of an increasing number of functional amyloids conferring bacterial colonies structural integrity and cohesiveness, like curli [121], suggested that prion‐like conformational transitions to amyloid structures might also exist in prokaryotes.

A bioinformatic survey of PrLD‐containing proteins in 839 different bacteria proteomes using PrionScan identified 2200 prion‐like candidates, accounting for a 0.3% of the analyzed dataset [28]. Strikingly, this percentage increases significantly when only pathogenic bacteria are considered. In *Staphylococcus aureus*, a virulent pathogen that is currently the most common cause of infections in hospitalized patients, predicted prion‐like proteins are 18% of the proteome. This suggests a role of PrLDs in pathogenesis, something consistent with the fact that many microbial functional amyloids are utilized by pathogens for invasion and maintenance of infection.

Regarding the biological processes, a Gene Ontology (GO) terms analysis of these PrLD‐containing proteins revealed an enrichment in cell morphogenesis (cell projection and cell wall dynamics), in secretion, in invasion and virulence, and in nucleotide metabolism. A survey of the Pfam families in such prion‐like proteins revealed an enrichment in nucleotide binding domains; however, they differ from those found in eukaryotes and include those in GTP‐binding elongation factors or the Rho termination factors. Other enriched Pfam domains are connected to cell wall dynamics, cell wall metabolism, attachment to the cell wall, and secretion and invasion processes. Therefore, as it occurs in eukaryotes, bacterial PrLDs seem to be constituents of proteins involved in the regulation of gene expression and transcription. The downstream processes they control differ significantly, playing essential roles in specific functions at the periphery of the cell, such as the establishment of cell contacts to form multicellular associations and biofilms [122, 123], which protect against stress and antibacterial agents, while facilitating the attachment to host cells, explaining why prion‐like proteins seem to be more prevalent in pathogens.

With the above data in hand, Pallarès *et al*. [60] combined composition‐based and soft amyloid analysis‐based approaches to scrutiny the prionic content of the Gram‐positive, anaerobic pathogen *Clostridium botulinum* pathogen, identifying 54 proteins that accounted for a 1.5% of the whole proteome. As in the previous study, one of the top ranked candidates was the Rho termination factor, which indeed contains a PrLD in > 20 different bacterial species. The Rho factor is a conserved hexameric helicase essential for the termination of transcription by RNA polymerase [124]. Although the prion activity of *C. botulinum* Rho could not be validated in its natural host, results obtained in vitro, in yeast and *Escherichia coli* clearly demonstrated its ability to populate amyloid‐based prion conformations, becoming the first prion‐like protein described in bacteria [30, 60, 63].

Soon after, the PrLD of the single‐stranded DNA‐binding protein (SSB) from *Campylobacter hominis* became the second experimentally validated bacterial prion domain [125]. This PrLD can populate two alternative conformations in *E. coli*, a soluble state without prion activity and an amyloid‐like prion conformation (SSB cPrD) that can be propagated over at least 100 generations.

When the evolutionary behavior of PrLD's in bacteria is analyzed in detail, a pattern of apparent sporadic conservation is observed, which is often coupled to a wide distribution across multiple phyla [126], with Rho and SSB prion‐like families displaying both features. This suggests that these domains are not necessarily needed to develop the primary function of the protein. Their presence may be occasionally beneficial, but eventually, they may become detrimental to the fitness of a given species and subsequently purged out. Alternatively, it could be that we are not detecting these domains simply because they present compositions that differ from those of the prion‐like domains used to train the algorithms. In this context, it should be noted that the algorithms used for the identification of prions in bacteria are trained on top of yeast prions sequences, while it has been described that bacteria generally have a lower proportion of Q/N‐enriched regions [25].

Overall, the studies detailed in this section provide support for the hypothesis that the presence of prion‐like proteins in the bacterial domain of life is not anecdotic, and they are likely a source of phenotypic diversity that allows adaptation to defying environments. However, many questions are still open, and much of bacteria's prionic landscape remains to be understood.

### The viral proteomes

If eukaryotic and bacterial cells bear prion‐like proteins, why not the viruses that infect them? This was the question Tetz and Tetz [34, 35] addressed by analyzing the prionic‐load of bacteriophages and eukaryotic viruses using PLAAC. They detected > 5000 and > 2500 putative prion‐like polypeptides in bacteriophages and eukaryotic viruses sequenced genomes, respectively. In eukaryotic viruses prion‐like candidates were more prevalent in DNA than in RNA viruses and in enveloped viruses relative to nonenveloped ones.

Functional annotation analysis evidenced an enrichment in virus‐specific processes involved in the establishment of interactions with the host cell. In the case of bacteriophages, the predominant functions corresponded to the attachment and penetration inside the bacteria. It remains unknown if these processes are also dependent on the presence of the peripheral prion‐like proteins predicted in bacteria. If this is the case, it will constitute a unique case of PrLDs mediated cross‐talk between different kingdoms of life. Not surprisingly, prion‐like proteins in eukaryotic viruses were related to the adhesion and entry of viral genetic material inside the host. Of course, a large fraction of PrLD‐containing proteins was involved in the binding and replication of nucleic acids, which against stems from the necessary association between these disordered regions and DNA/RNA binding domains.

Overall, PrLD‐mediated switching on/off of regulatory proteins seems to be a generic and conserved mechanism, whereas the activated/inactivated process or function would be specific for any particular kind of organism. Consistent with this view, Nan and co‐workers reported the first viral protein that behaves as a prion [127]. The AcMNPV virus‐encoded LEF‐10 protein works as a late expression factor essential for tDNA replication and gene expression [128]. Its prion behavior has been corroborated in yeast and in the natural host, insect cells. Inside its native host, LEF‐10 can populate two different conformations, a soluble and active one, and an aggregated and inactive state that results in a reduction of late gene expression. This conformational conversion is highly dependent of the number of virions in the host cell, in such a way that LEF‐10 acts as a self‐limiting factor becoming inactive when the presence of new virions is sufficient. It is worth to mention that the LEF‐10 prion domain does not conform to the conventional conformational bias, escaping from the identification of the above‐described predictors. This is not an exception since several well‐characterized prions lack these domains [9], which argues for the development of more accurate algorithms since otherwise a significant fraction of the prion sequential space would remain obscure.

Viruses behave as obligate parasites, in such a way their survival and replication in host cells requires adaptation to cellular conditions. The use of prion mechanisms for a fast regulation of gene expression might help viruses to quickly respond to the response to their presence elicited by host. Hence, we anticipate that more viral prion‐like proteins will be discovered soon and indeed a recent study suggests that a PrLD in the SARS‐CoV‐2 spike protein might influence the affinity of this viral protein for angiotensin‐converting enzyme 2 in human host cells [129].

## Conclusions

The proteome studies discussed in this review make clear that the list of identified proteins bearing PrLDs will be steadily increasing and that they are widespread in all life forms, even in viruses. As a trend, PrLDs function as gene expression regulatory devices; however, these predictions have unveiled unprecedented and fascinating examples of prion‐mediated physiological functions. PrLDs emerge as proteinaceous entities that encode molecular information essential to ensure complex cellular events such as cell cycle regulation, immune response, inflammation, long‐term memory, circadian clock regulation, host adaptation, cell adaptability, and invasion, these latter functions being more prevalent in pathogens.

Proteins hosting PrLDs are modular and multifunctional polypeptides that establish both homotypic and heterotypic protein–protein interactions, preferentially with other PrLDs. It is likely a synergy between these features that links PrLDs to their biological functions. The transient and weak noncovalent interactions that allow PrLDs to sample different conformational states appear to overlap somehow with those responsible for the liquid−liquid phase separation of different biomolecular condensates. Mutations in these regions that impact these contacts' strength/number can trigger a transition from highly dynamic liquid condensates to more solid‐like aggregates, abrogating their dynamic/promiscuous nature, leading to the onset of devastating protein aggregation diseases. From an evolutionary point of view, the presence of these inherently risky domains in multiple species across kingdoms is likely the result of a positive natural selection. Bioinformatics approaches have been invaluable in their identification and characterization. Nonetheless, prion‐like proteins that cannot be identified by compositional‐ and/or soft amyloid‐based PrLD predictors have been described [104, 105, 106]. These nonamyloidogenic proteins illustrate the co‐existence of different flavors of prions and challenge our knowledge of the compositional/sequential determinants of beneficial and pathological prion‐like transitions. An overlooked possibility is that the sequence features enabling prion‐like behavior could exhibit a significant degree of species‐specificity. In this scenario, a low Q/N‐content in any given species would be immediately translated into a decreased PrLD predicted content [25], independently if this true or not. Further experimental characterizations in novel organisms are needed to fuel novel prion‐like predictive strategies that flee from our present yeast‐centric view.

This is important not only because they will allow us to deep into the biology of the prion‐like proteins but also for their potential therapeutical and nanotechnological implications. In this last respect, the knowledge we have gained from proteome‐wide analyses has already crystallized in the development of new nanomaterials with fascinating functional properties [130, 131] that would have never seen the light without the herein described bioinformatic analysis.

## Conflict of interest

The authors declare no conflict of interest.
